# The WASH Approach: Fighting Waterborne Diseases in Emergency Situations

**DOI:** 10.1289/ehp.123-A6

**Published:** 2015-01-01

**Authors:** Wendee Nicole

**Affiliations:** Wendee Nicole has written for *Discover*, *Scientific American*, and other magazines.

To report this story, Wendee Nicole visited two refugee settlements in Northern Uganda, Arua District’s Rhino Camp and the settlements of Adjumani District. She celebrated Global Handwashing Day 2014 with dozens of young children at Rhino Camp.

“No water!” a young mother with short cornrowed hair says in her limited English, a worried look etched in her brow. She points to the spigots, dripping with the scant water remaining in the pipes.

**Figure:**
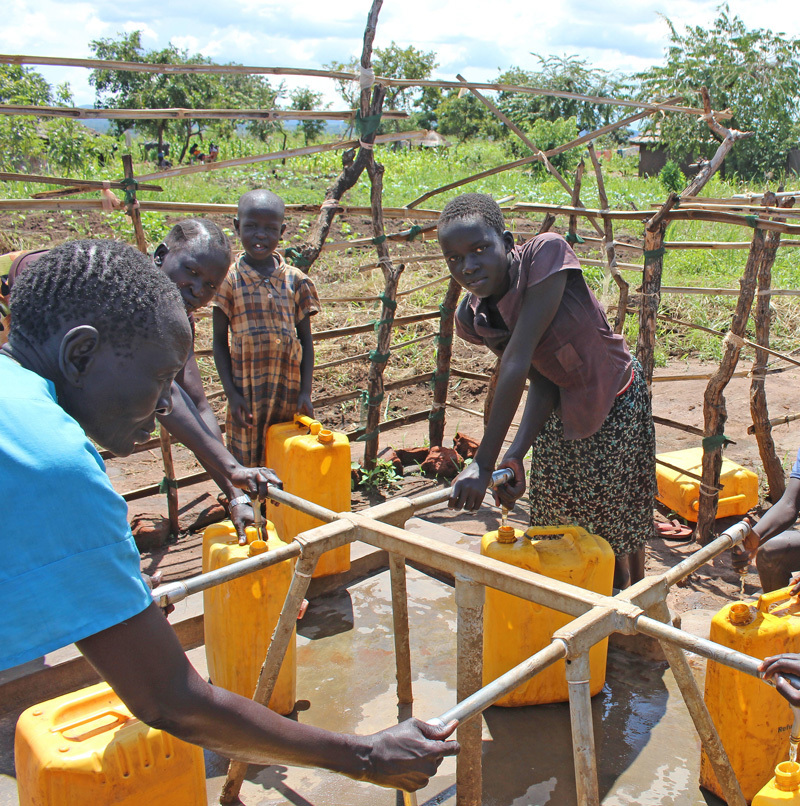
Refugees collect water from a public tap stand in an Adjumani settlement. © Wendee Nicole

It’s a sweltering October day just 3 degrees north of the Equator in Northern Uganda’s Adjumani District, where a dozen children and a handful of women have gathered at the public tap stand with their 20-liter jerrycans. They are among nearly 600,000 refugees who have walked hundreds of miles from South Sudan to neighboring countries, fleeing violence that began in December 2013.^1^ More than 66,000 men, women, and children have settled here, in camps in Adjumani District, with over 15,000 more in Arua District’s Rhino Camp.^2^

The tap stand has six spigots that, until a few days ago, supplied an ample supply of fresh water. “It was producing 140,000 liters of clean water, but two weeks ago the pump malfunctioned,” explains Tim Sutton, Oxfam’s WASH (Water, Sanitation, and Hygiene Promotion) interim team leader for the Adjumani and Arua settlements. Working under the aegis of the United Nations High Commission on Refugees (UNHCR) and Uganda’s Office of the Prime Minister Department of Refugees, Oxfam installed an electric generator in June 2014 that provides water for two of six refugee settlements in Adjumani District.

**Figure:**
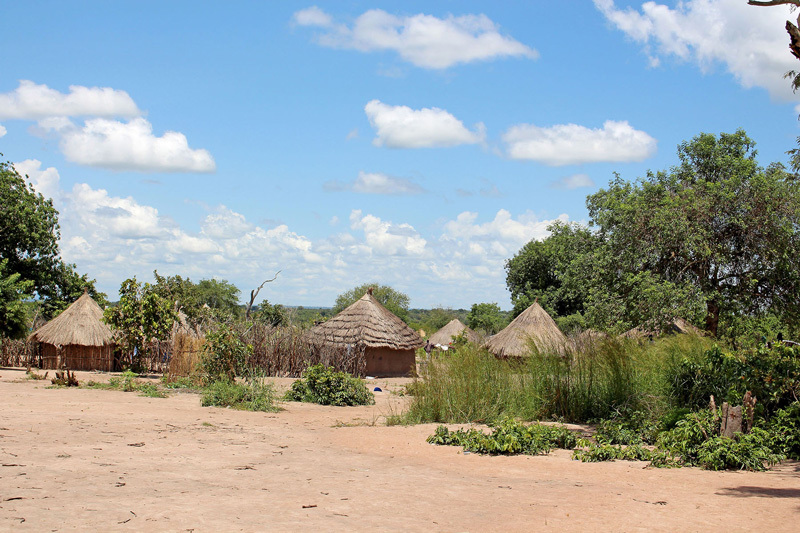
Rhino Camp, Arua District. Refugees in Uganda live on land donated by Ugandan nationals. Refugee families are given plots on which they can build temporary shelters and grow crops. © Wendee Nicole

Although the refugees can still get water from hand-operated pumps on other boreholes, accessing them requires walking further distances and physical labor to prime the pump. Sutton reassures the young mother, his own brow furrowed with concern, “We are working on it.”

Sutton, who has engineered solutions for humanitarian crises around the world, knows well the importance of ready access to clean water. Most pressing, of course, is the need for drinking water. But the lack of water for washing also puts people’s lives at risk from waterborne illnesses spread by the fecal–oral route.

Fecal–oral diseases can proliferate rapidly, sometimes to epidemic proportions, when people in crowded conditions lack clean water for hygiene and sanitation.^3^ Among the agents involved are at least 20 viral, bacterial, and protozoan pathogens that cause diseases such as cholera, bacillary dysentery, and the relatively recently discovered hepatitis E.^4^^,^^5^  Aid groups are combating these pathogens with WASH, an integrated approach to disease prevention that ensures not only that people in emergency situations have water and sanitation infrastructure, but also that they practice behaviors that prevent disease.

## The Threat of Diarrheal Diseases

Diarrhea may not seem deadly to Westerners who have access to improved sanitation. Nevertheless, it kills three-quarters of a million children every year, more than malaria, AIDS, and measles combined.^6^ The chief danger of diarrheal diseases is the loss of bodily fluids. “You get very weak within hours, and in fact you die of dehydration,” Sutton says. Worldwide, diarrheal diseases are the second leading cause of death of children under age 5 years^6^ and cause 40% of child deaths in the early stages of a humanitarian emergency, sometimes more.^7^

**Figure:**
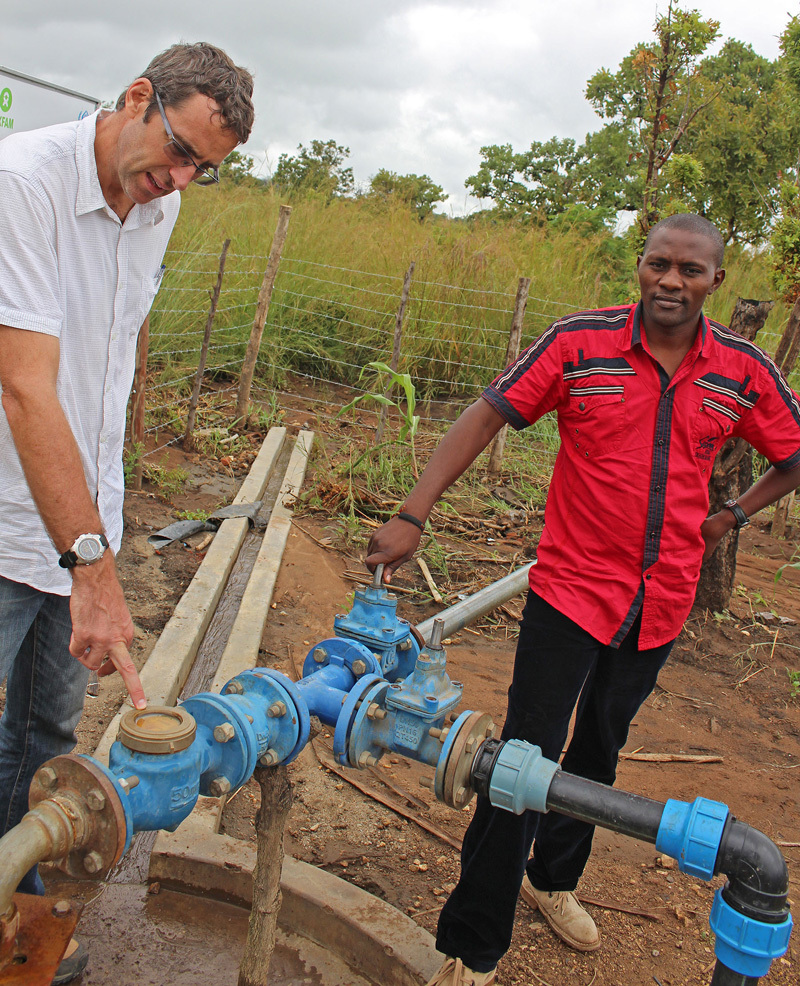
Oxfam staff members Tim Sutton (left) and Pius Nzuki Kitonyi (right) with the soon-to-be-repaired water pump in Adjumani. In disaster-affected situations, Oxfam takes a lead in delivering WASH-related services. © Wendee Nicole

Disaster situations are prime settings for disease outbreaks: Limited water tends to go first to drinking and cooking, while hygiene gets short shrift, especially among people who are just being taught the connection between hygiene, sanitation, and health.^8^ “If it’s too far to walk, people will only collect a very small amount of water,” says Sutton. “They’ll use it for drinking and cooking and won’t have enough water to practice good hygiene.”

But access to water isn’t enough; health-protective behaviors are critically important. Worldwide, only 19% of people on average are estimated to wash their hands with soap after defecating (see table).^9^ Yet studies consistently show that handwashing with soap is effective at reducing diarrheal diseases; one systematic review of the literature estimated it reduces risk by 23–40%,^9^ while another estimated a 48% reduction.^10^

Although workers can install latrines and teach the value of handwashing and latrine use, only the refugees themselves can choose to change their behaviors. And that means changing social norms. Open defecation is common practice in developing nations. Handwashing is often done without soap,^8^ and cultural traditions such as eating with the hands and sharing plates can spread infectious diseases.^11^

“You need to understand the ‘F diagram,’” says Sutton, referring to the traditional schematic that sketches out all the fecal–oral disease transmission pathways (see figure). Feces, and whatever infectious agents are contained within it, can spread through fluids, fingers, flies, and the fields in which people defecate and/or grow crops. By not washing properly or keeping food in hygienic conditions, fingers, fluids, and flies can also contaminate food.^11^

Drinking, bathing, and cooking with unclean water sources can cause illness as well. Surface waters are likely to be contaminated with fecal pathogens, especially during the rainy season when rainwater washes feces into waterways.^12^

## A “New” Hepatitis

Poor hygiene and fecal contamination were major factors in one of the world’s biggest outbreaks of hepatitis E, which began in October 2007 and persisted for a couple of years.^13^ This outbreak affected camps for internally displaced persons (IDPs) in Northern Uganda’s Kitgum District, infecting more than 10,000 people and killing 160, mostly pregnant women and young children. Other recent hepatitis E outbreaks have occurred among refugees and IDPs in Kenya, South Sudan, and Chad.

The term “hepatitis” refers to liver inflammation, most commonly caused by unrelated viruses A, B, C, D, and E as well as other hepatropic viruses. Hepatitis E is an emerging pathogen that, like hepatitis A, spreads by the fecal–oral route. It is particularly dangerous for pregnant women, especially in the third trimester. Recent data suggest hepatitis E could be responsible for approximately 10% of maternal deaths in Bangladesh.^14^

Hepatitis E was first described as “non-A, non-B hepatitis” in 1980.^15^ During a 1983 outbreak in Afghanistan, researcher Mikhail Balayan purposely exposed himself to the hepatitis E virus (HEV) to determine the causative agent of the illness. According to one account, “Though he wanted to bring samples back to his Moscow laboratory, he lacked refrigeration. So he made a shake of yogurt and an infected patient’s stool, drank it, went back to Moscow, and waited. When he became seriously ill a few weeks later, he started collecting and analyzing his own samples.”^16^

Balayan detected the novel virus by electron microscopy,^17^ and the single-stranded RNA virus was characterized in 1989 and sequenced in 1991.^18^ Hepatitis E is more common in the tropics and subtropics, particularly Asia and Africa. (Less relevant to refugee settlements, a swine HEV genotype that also infects humans is associated with illnesses in the industrialized world. This strain is transmitted by consumption of raw or undercooked pork sausage.^19^ There is evidence that incidence of HEV infection in industrialized nations is increasing.^20^^,^^21^)

Oxfam public health promoter Tracy Lamwaka trained and supervised volunteers in the Kitgum camps at the time of the outbreak there. “There was a lot of open defecation taking place,” Lamwaka says. “The water sources were also insufficient, and there was very poor food handling. There were so many people within the camp, it was very easy for [HEV] to spread.”

**Figure:**
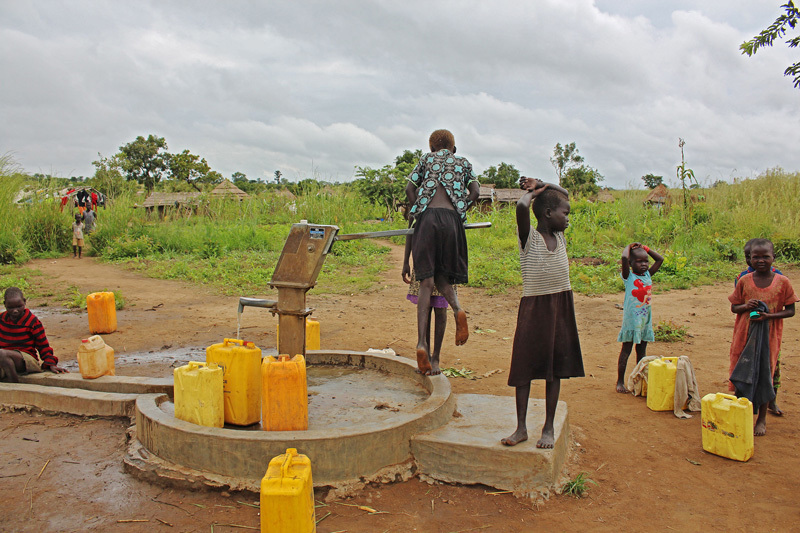
Hand-operated water pumps are a reliable source of precious water but require physical labor to get the water flowing. © Wendee Nicole

Oxfam engaged in hygiene education campaigns in the camps, training IDPs as “hygiene promoters” who educated their neighbors and kept an eye on sanitation in their camps. Each promoter monitored 20 households—checking, for instance, on whether people were washing their hands and utensils and whether anyone showed disease symptoms. The spread of HEV was brought under control but then spiked a second time.^22^

Until the Kitgum outbreak, most researchers believed HEV spread only through drinking contaminated water. But evidence from Kitgum suggested the virus may also spread directly from person to person. Eyasu Teshale, an epidemiologist with the U.S. Centers for Disease Control and Prevention, and colleagues reported in 2010 that “investigation did not reveal a clear continuing common source of infection necessary to sustain the epidemic for many months. Chlorination of drinking water had been implemented early in the epidemic, … and HEV RNA was not detected in borehole well water samples or from the nearest alternative source of drinking water.”^22^

**Figure:**
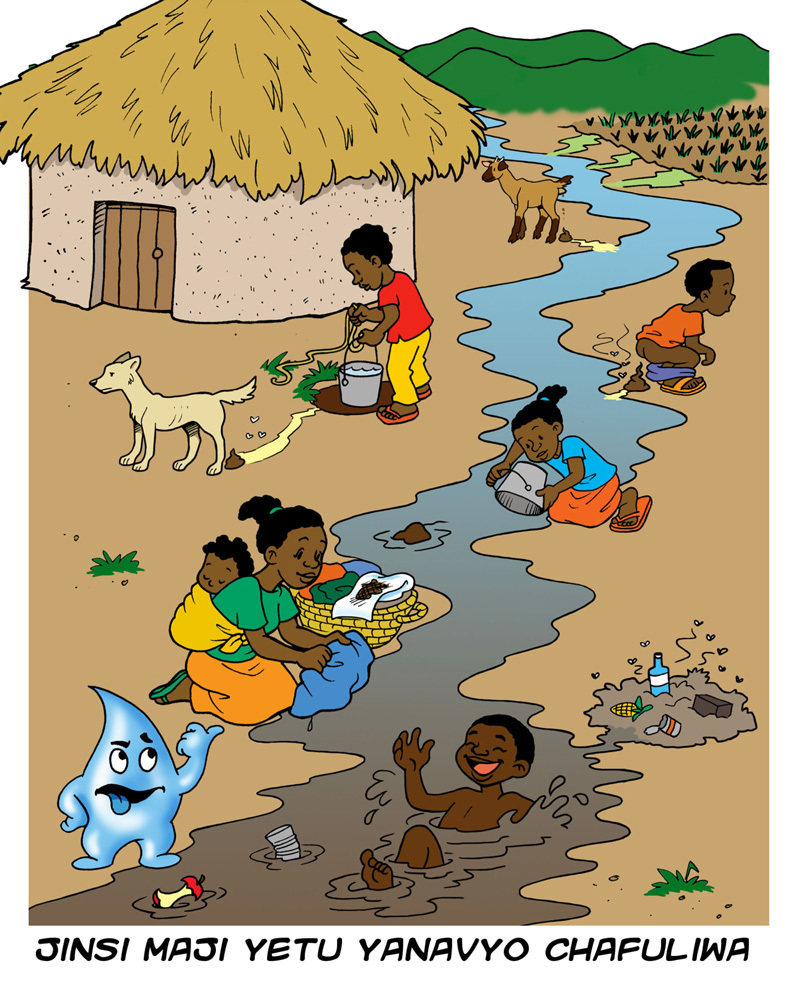
The Swahili caption of this poster reads “How our water becomes contaminated.” This and other educational materials are available in multiple versions, customized for the various regions where open defecation is commonplace, including Africa, Latin America, the Caribbean, and South, South East, and South West Asia. Source: Centre for Affordable Water and Sanitation Technology/http://resources.cawst.org/

If there were no secondary modes of transmission, Teshale explains, this outbreak would have been controlled with provision of safe and adequate drinking water. But it’s also too soon to definitively attribute cases to person-to-person transmission, he says.

If person-to-person transmission were to occur, it could potentially make hepatitis E much harder to contain. Rather than someone needing to drink contaminated water, they would only need to come in contact with a family member or a shared utensil carrying the virus.

“Our findings^22^ are from a unique setting, which since then has become a common setting for hepatitis E outbreaks in sub-Saharan Africa,” Teshale says. People in IDP or refugee camps are living in crowded conditions with poor environmental and household hygiene and inadequate water supply. “[T]he outbreak is sustained by the ongoing fecal contamination of water, food, water-collecting vessels, water and food, serving utensils, or the hands within the infected persons’ personal, social, or household contacts,” Teshale says. “Fecal excretion of HEV during illness is very high, allowing higher contamination risk.”

**Moving Beyond Camps**

Refugees in settlement camps are supposed to stay put, but some return to their home country to visit family, or travel to cities. This can increase the risk of spreading disease. If a refugee comes down with a highly contagious and deadly disease, such as Ebola or Marburg, such movement could prove tragic—and the risks are real. In September 2014 a Ugandan died of Marburg hemorrhagic fever in a Kampala hospital,^33^ and in August 2014 an Ebola outbreak separate from the ongoing West Africa epidemic arose in Democratic Republic of Congo, killing 49.^34^ The U.S. Centers for Disease Control and Prevention declared the country Ebola-free on 21 November 2014.

Although Ebola and Marburg are not fecal–oral diseases per se, they can spread rapidly when people do not engage in hygienic behaviors, and as with any emergency situation, stopping an epidemic’s spread will ultimately depend on changing behaviors, and quickly. As one recent review stated, “The success of interventions to improve WASH practices ultimately rests on the ability to foster and maintain behaviour change at the individual, household, community, and structural levels.”^35^

## A Humanitarian Crisis

The broken pump at Adjumani is upsetting for the residents, and if it’s not fixed soon, the situation could rapidly develop into a health crisis. “It’s very serious,” Sutton says. But it is a relatively small setback compared with the humanitarian crisis in Kitgum or the situation a couple of years ago in Adjumani itself, when refugees first started arriving.

“If you had been here at the beginning, it would have been very different,” says Sutton. “There were just tens of thousands of people. People were living outside. Land hadn’t been allocated to households. These camps are in the middle of the bush.”

Refugees are most vulnerable to waterborne diseases in the first stage of a crisis. “They’ve walked from South Sudan. Maybe the children are suffering a bit of malnutrition. There are no toilets. They’re doing a lot of open defecation,” says Sutton. “They’ve got no access to clean water or not enough access to clean water to practice good hygiene and to do basic domestic duties. So this is a precarious situation. You’ve got to get water to people immediately.”

**Figure:**
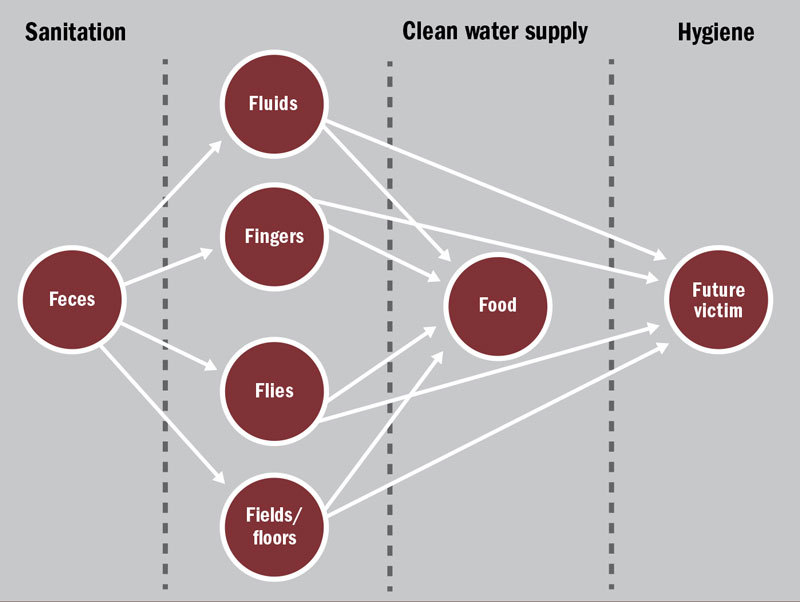
The pathways of fecal–oral transmission all start with “F,” hence the name “F diagram.” Water, sanitation, and hygiene act as barriers to prevent contact with feces. Joseph Tart/EHP

Crisis management practices have changed over time. “Back in the eighties, we used to look at water on its own, and we used to look at sanitation on its own,” Sutton says. “And then we realized to have more health impact … we’ve got to start looking at things as a package.” The integrated WASH approach was born.

Oxfam and many other nonprofit entities adhere to the Sphere Project standards, which specify minimum requirements for water, sanitation, and shelter in humanitarian crises.^23^ Under these standards, people should have 7.5–15 liters of water per person per day for drinking, cooking, and hygiene; at a bare minimum, they should have 2–3 liters/person/day for drinking and food preparation. In Adjumani, the goal is to reach UNHCR’s higher standard of 20 liters/person/day^24^ within a couple of months, says Thurein Maung, the UNHCR WASH coordinator for the district.

In disaster-affected situations, Oxfam takes a lead in delivering WASH-related services. Often the first phase is hauling treated water to each settlement and filling large tanks from which refugees get water. Organizations also employ what they call a “sanitation ladder.” At the start of a crisis, the first step is to contain the spread of feces. Initially agencies may allocate specific fields or trench latrines for defecation. In addition, aid agencies may promote the so-called cat method, encouraging people to bury their feces. Moving up the next rung of the “ladder,” aid agencies install emergency slab latrines, often a plastic slab incorporating a drop hole that can be covered to keep flies and odor to a minimum.^25^ “Tippy taps” made of a jerrycan, some sticks, string, and a bar of soap make simple but effective handwashing stations.^26^

## Getting People to Use Latrines

Teaching people to use latrines, wash with soap, or engage in other new behaviors takes time, says Oxfam public health engineer Pius Nzuki Kitonyi. “First we have a meeting so that we can hear their [views and priorities]. Then from there, we give them the information—‘This is what we intend to do.’ If it’s new, we tell them, ‘This will improve on what you have been doing.’ And then we start implementing.” Ideally, refugees themselves do some of the work, digging holes for latrines, trenches for water pipes, or clearing land for roads, in “pay for work” schemes that allow them to achieve some sort of socioeconomic independence, according to Oxfam.

In practice, getting people to use latrines—or other new behaviors—is hard when they have grown up with their own cultural beliefs. “Most of the [refugees], they … go to the bush and do their stuff there,” explains Wilson Senyonyi, an Oxfam protection officer. “When pregnant mothers go to the latrines, they fear the child will just come out and fall into the pit.” These and other cultural beliefs can keep people from using modern latrines.

One technique used by UNHCR and its partners in refugee situations is called the Community-Led Total Sanitation (CLTS) approach.^27^ There are different steps involved in CLTS, including what Maung calls “the calculation of s—t.” In this exercise, a facilitator discusses how many grams of feces is produced by one person per day. The facilitator asks participants how many people there are and then calculates the total amount of feces in people’s living environment for a year or two.

Next, Maung says, “We ask them where those feces are. Before this exercise, the facilitator also walks with people through a community and identifies open defecation around the dwelling. We show them ‘you are living in s—t.’ It makes them uncomfortable. We bring a sense of shock or shame, while respecting the culture.”

**Figure:**
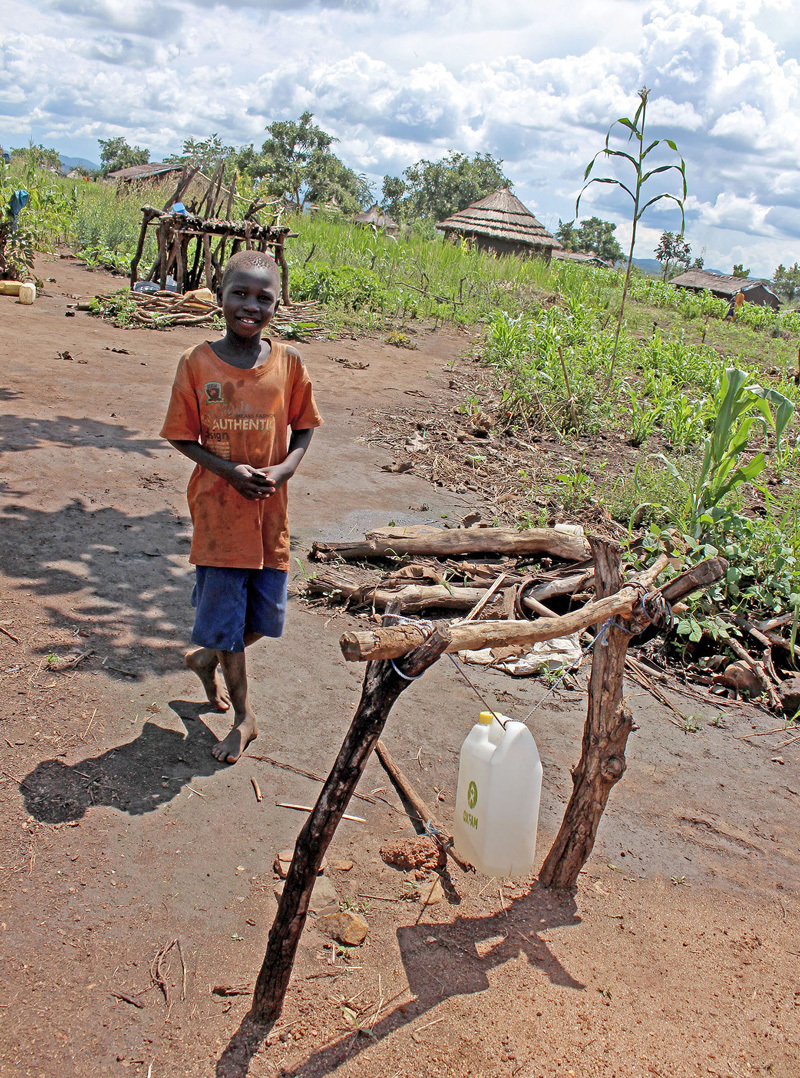
The foot-operated “tippy tap” provides a low-cost, low-tech, hands-free handwashing station. © Wendee Nicole

The goal is to ignite awareness and a genuine desire for change in the community, and it works, Maung says—typically within a week people will begin building latrines or start using ones that are already there. But the effectiveness of CLTS largely depends on the skill of the facilitator, he adds.

**Table 1:** Globally, only 19% of people are estimated to wash their hands with soap after defecating, according to a systematic review of 42 studies on handwashing prevalence from around the world.^9^

Country	Number of studies	Estimated prevalence (%) (95% confidence interval)*
Burkina Faso	1	8 (4, 14)
Ethiopia	1	22 (13, 34)
Ghana	3	13 (6, 22)
Kenya	5	15 (7, 29)
Senegal	1	19 (12, 30)
Uganda	1	15 (9, 24)
Tanzania	1	5 (3, 10)
USA	7	49 (32, 65)
Peru	2	16 (7, 32)
Israel	1	12 (5, 26)
Netherlands	1	50 (34, 66)
United Kingdom	3	52 (34, 70)
Kyrgyzstan	1	16 (7, 32)
Bangladesh	7	18 (10, 27)
India	3	15 (3, 27)
Thailand	1	25 (15, 38)
New Zealand	1	72 (44, 89)
Republic of Korea	1	17 (9, 33)
China	2	13 (6, 24)
*Studies were not intended to be country-representative. Country averages were therefore estimated without weighting by sample size.		

In typical development projects, the activity requires people to use their own locally available building materials so they become invested in the process. Refugees have a different situation, however, so the process is adapted slightly. “Traditionally we don’t provide materials to construct the latrines, but in refugee situations, we create demand first, then provide materials,” Maung says; this encourages the refugees to use the facilities.

But does the mere presence of latrines and improved infrastructure really improve health outcomes? A team of researchers studied 100 villages in Odisha, India, some of which received latrines and some of which did not. They found no significant differences in diarrheal incidence or associated deaths, fecal contamination of water stored in households, contamination of the hands of mothers and children, malnutrition, or helminth worm infection between villages with and without additional latrines.^28^

“The lesson to draw is not that latrines don’t produce a benefit but the latrine programs weren’t properly implemented,” says Sandy Cairncross, a professor of environmental health at the London School of Hygiene & Tropical Medicine, who was not directly involved in the Odisha study. Public health improvement depends on changing people’s contact with to feces, he explains. And that requires changing behavior.

## Encouraging Behavioral Change

Since the 1990s Cairncross and his collaborator at London School of Hygiene & Tropical Medicine, epidemiologist Val Curtis, have built their careers advancing the scientific basis for health education. Curtis calls herself a “disgustologist,” and her work focuses on understanding how health behaviors can be effectively changed. Specifically, she has shown that disgust is effective at getting people to avoid behaviors that contribute to disease.^29^

Curtis and Cairncross’s research has been funded, in part, by Unilever, which allowed them to bring private-sector marketing and advertising know-how to studying how best to promote health-protective behaviors.^30^ “Some whitecoat giving lectures to mothers with crying babies isn’t going to make an impact on anyone at all,” says Cairncross. “Telling people if they didn’t wash their hands they’ll get diarrhea and die—these doom-laden negative messages related to the threat to someone’s health don’t stop people doing unhealthy things or get them to do healthy things.”

**Figure:**
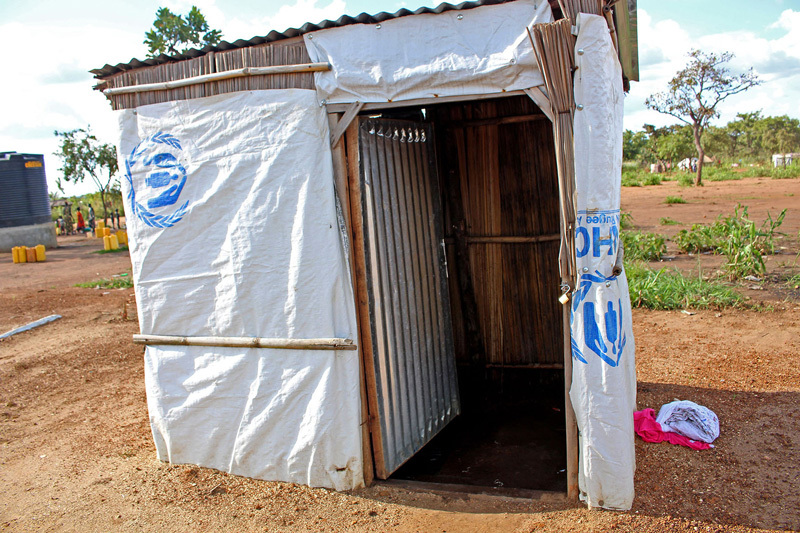
Latrines can be a drastic and unwelcome departure for people who are accustomed to defecating in the open, and the public health benefits are not necessarily easy to sell. © Wendee Nicole

On the other hand, he says, positive messages such as “If you wash hands with soap you’re a good mother” or “This is what all modern people are doing nowadays” or “It makes your hands taste nice when you eat your rice with your hands” are much more likely to inspire behavioral changes. “It’s not rocket science,” he says. “Advertisers have been doing this for years.”

But people also need repeat messages. Generally speaking, Cairncross says, people need to hear about washing their hands with soap about six times in a month before the message sticks.

To raise awareness among people in developing nations, the Global Public–Private Partnership for Handwashing with Soap started a campaign that includes Global Handwashing Day, celebrated October 15 around the world.^31^ At Arua’s Rhino Camp, dozens of children and their parents gathered to celebrate Global Handwashing Day 2014. Girls and boys sang songs and dramatized the importance of handwashing at critical times such as after using the toilet and before eating, as well as washing one’s head, feet, and body. Everyone marched around the open field before enjoying sodas and water—after washing their hands with soap. Community leaders from within the refugee settlements led the training of the children, along with various groups such as Oxfam and UNHCR.

## Stabilizing the Crisis

With several months of hard work since the refugees began arriving here, the Adjumani and Arua settlements resemble traditional African villages. Latrines, boreholes, and roads have been built, and families have been assigned plots of land they can temporarily call their own. Before long, technicians repair the broken pump, and water soon returns to tap stands throughout the settlement.

Unlike a traditional refugee setting, where refugees live in tents in a fenced-off camp, those in Uganda live on land donated by Ugandan nationals through arrangement of the government, and refugee families are given plots on which they can build shelters and grow crops.^32^ The Ugandan government and UNHCR have a policy of devoting 70% of aid to refugees and 30% to nationals, according to Maung. This arrangement helps reduce resentment and also improves infrastructure that remains once refugees return home.

**Figure:**
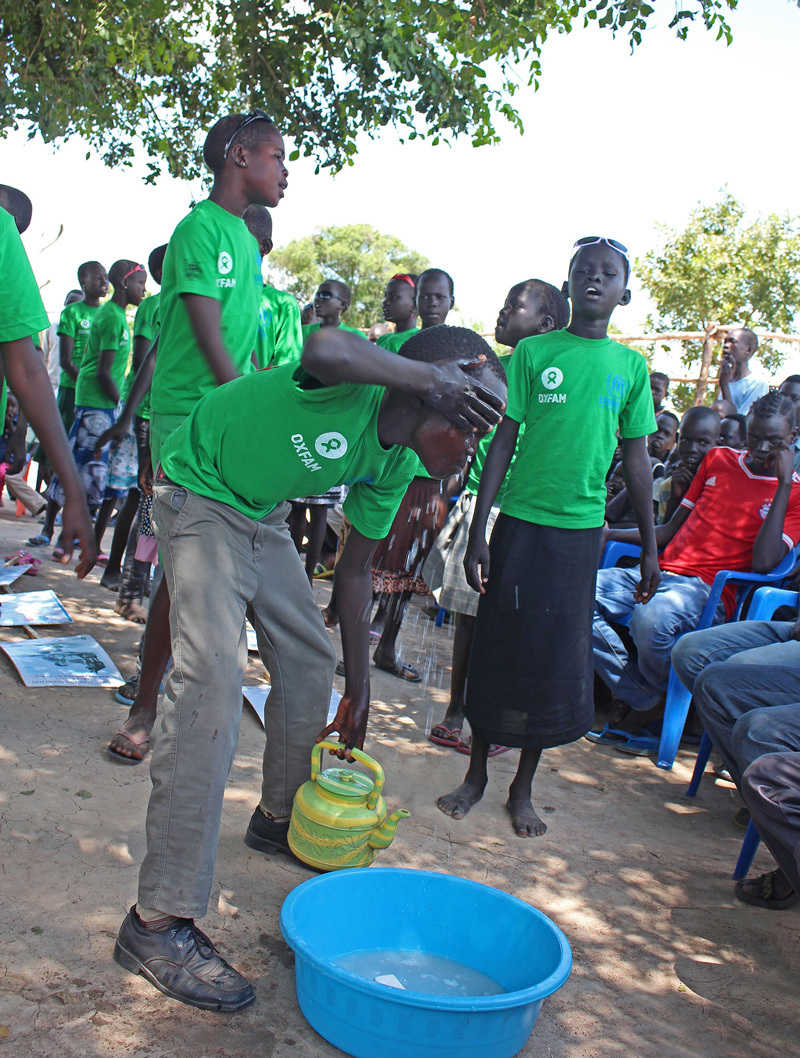
The children of Rhino Camp celebrated Global Handwashing Day with songs, demonstrations, and a parade. © Wendee Nicole

“We are in the process of transition from acute emergency to stabilized emergency,” says Maung, “but [there’s] quite a lot of work to be done in terms of operation and maintenance of WASH facilities to ensure sustainability.”

“There is not much data on long-term behavior change for handwashing with soap,” says Jelena Vujcic, an epidemiology researcher at the University of Buffalo. Outstanding questions include how to make handwashing with soap a social norm in a way that’s culturally conscious and acceptable to a society, and which handwashing technologies support good handwashing practice and are acceptable to the communities that use them.

“Overall, there is a dearth of data on handwashing in emergencies,” Vujcic says. “There is no evidence on what works in emergencies to improve handwashing behavior.” But with no shortage of emergencies for UNHCR to attend to around the world, both humanitarians and epidemiologists will have their hands full for the foreseeable future—and hopefully those hands will be cleaned with soap.
